# Prophylactic Oral Application of Activated Charcoal Mitigates Acute Campylobacteriosis in Human Gut Microbiota-Associated IL-10^−/−^ Mice

**DOI:** 10.3390/biom14020141

**Published:** 2024-01-23

**Authors:** Markus M. Heimesaat, Niklas Schabbel, Luis Q. Langfeld, Nizar W. Shayya, Soraya Mousavi, Stefan Bereswill

**Affiliations:** Gastrointestinal Microbiology Research Group, Institute of Microbiology, Infectious Diseases and Immunology, Charité—Universitätsmedizin Berlin, Corporate Member of Freie Universität Berlin, Humboldt-Universität zu Berlin, and Berlin Institute of Health, 12203 Berlin, Germany

**Keywords:** activated charcoal, enteropathogenic infection, *Campylobacter jejuni*, immune-modulatory effects, secondary abiotic IL-10^−/−^ mice, human gut microbiota-associated mice, campylobacteriosis model, host-pathogen interaction, placebo-controlled pre-clinical intervention study, prophylactic treatment

## Abstract

The incidence of human *Campylobacter jejuni* infections is increasing worldwide. It is highly desirable to prevent campylobacteriosis in individuals at risk for severe disease with antibiotics-independent non-toxic compounds. Activated charcoal (AC) has long been used as an anti-diarrheal remedy. Here, we tested the disease-mitigating effects of oral AC versus placebo in human gut microbiota-associated (hma) IL-10^−/−^ mice starting a week prior to *C. jejuni* infection. On day 6 post-infection, the gastrointestinal *C. jejuni* loads were comparable in both infected cohorts, whereas campylobacteriosis symptoms such as wasting and bloody diarrhea were mitigated upon AC prophylaxis. Furthermore, AC application resulted in less pronounced *C. jejuni*-induced colonic epithelial cell apoptosis and in dampened innate and adaptive immune cell responses in the colon that were accompanied by basal concentrations of pro-inflammatory mediators including IL-6, TNF-α, IFN-γ, and nitric oxide measured in colonic explants from AC treated mice on day 6 post-infection. Furthermore, *C. jejuni* infection resulted in distinct fecal microbiota shift towards higher enterobacterial numbers and lower loads of obligate anaerobic species in hma mice that were AC-independent. In conclusion, our pre-clinical placebo-controlled intervention study provides evidence that prophylactic oral AC application mitigates acute murine campylobacteriosis.

## 1. Introduction

Human infections with the zoonotic enteropathogen *Campylobacter jejuni* are increasing all over the globe and are responsible for serious morbidities and tremendous health care and socioeconomic expenses [1]. In fact, *C. jejuni* constitutes the most frequently reported food-borne bacterial infectious agent of enteritis worldwide [1,2]. In Europe, more than 127,000 campylobacteriosis cases were reported in 2021 [3]. The main reservoir of the thermophilic and highly motile Gram-negative rod bacteria is the intestinal tract of warm-blooded vertebrates, where *Campylobacter* reside as part of the commensal gut microbiota without causing harm to the host [4,5]. Humans become infected following ingestion of undercooked contaminated meat products, particularly from poultry, and surface waters, for instance [6]. After an incubation period of approximately 3–7 days, infected humans complain about enteritis symptoms including general malaise, bloating, abdominal cramps, nausea, vomiting, watery or even bloody diarrhea with mucous discharge, and fever [7,8]. Following survival of the gastroduodenal passage, *C. jejuni* adhere to and invade the intestinal epithelial cells and transmigrate further to the subepithelial tissues [9,10]. Subsequently, Toll-like receptor-4 (TLR-4) dependent sensing induced by the *C. jejuni* cell wall constituent lipo-oligosaccharide (LOS) leads to recruitment of both innate and adaptive immune cells, such as macrophages, monocytes, neutrophils, and T lymphocytes, to the infected colonic mucosa and lamina propria [11]. The pro-inflammatory mediators released by the stimulated immune cells do not only aim to limit the enteropathogenic infection, but also result in severe intestinal tissue damage such as cellular apoptosis and ulcerations resulting in a compromised epithelial barrier and malabsorptive “leaky gut disease” [12,13,14]. In most cases, the campylobacteriosis symptoms resolve without residues within two weeks post-infection (p.i.). With a latency of several weeks or even months after the initial enteritis event, however, autoimmune diseases can occur on rare occasions [15]. These post-infectious complications might affect the nervous system (e.g., Guillain-Barré syndrome (GBS)), the joints (e.g., reactive arthritis), and the intestinal tract (e.g., irritable bowel syndrome, inflammatory bowel diseases) [16,17,18]. Importantly, the risk for the development of such post-infectious collateral damages of *C. jejuni* infection directly correlates with the severity of the preceding enteritis [19]. Campylobacteriosis patients are usually treated symptomatically with non-steroidal analgetics, antipyretics, and replacement of fluids and electrolytes [8]. In severe and invasive courses with bacteremia in immunocompromised patients, however, antibiotic treatment with macrolides and fluoroquinolones might be additionally indicated [8,20]. One needs to take into consideration that the emergence of multi-drug resistant *Campylobacter* strains could hamper successful treatment of severely diseased patients [21]. Therefore, it is highly significant to identify antibiotic-independent treatment options to mitigate human campylobacteriosis and, thereby, to decrease the risk for post-infectious sequelae afterward.

Activated charcoal (AC) has been used as anti-diarrheal agent for centuries [22]. AC is usually prepared from plant material containing carbon and activated by heating. It possesses large pores and provides a large surface area and, hence, favorable prerequisites for the absorption of distinct molecules providing harm to the vertebrate host. AC has long been used as an antidote for the treatment of intoxications [22] and of infectious enteritis caused by *Escherichia coli*, *Vibrio cholerae*, and *Cryptosporidium* species [22,23,24,25]. In clinical studies, AC was shown to mitigate diarrhea caused by antibiotic and chemotherapeutic agents [26,27,28] and to alleviate symptoms of patients diagnosed with irritable bowel syndrome [29,30]. Notably, AC was shown to exert anti-inflammatory effects due to the binding and inactivation of bacterial endotoxins including LOS and of pro-inflammatory mediators [31], making AC an attractive candidate in the combat of acute campylobacteriosis. In order to address this in a pre-clinical setting, we applied IL-10^−/−^ mice harboring a human gut microbiota as a *C. jejuni* infection and inflammation model [32]. Importantly, conventional laboratory mice are protected from *C. jejuni* infection due to the host-specific composition of the murine gut microbiota [33]. This protective colonization resistance can be overcome by gut microbial depletion upon broad-spectrum antibiotic pre-treatment [33]. Remarkably, the colonization resistance can be sufficiently restored upon reintroduction of the complex gut microbiota from murine as opposed to human donors following oral fecal microbiota transplantation (FMT) [33]. This clearly indicates that the host specificity of the gut microbiota determines the resistance against and susceptibility towards enteropathogenic infection [34]. Notably, rodents including mice are approximately 10,000 times more resistant against TLR-4 ligands such as lipo-polysaccharides (LPS) and LOS [35], whereas *il10* gene deficiency can render mice susceptible to *C. jejuni* LOS [36]. In consequence, we use human gut microbiota-associated (hma) IL-10^−/−^ mice to dissect the triangular relationship between the enteropathogen *C. jejuni* on one side and the immune system and human gut microbiota on the other, the host side [32]. Within less than a week following oral *C. jejuni* infection, hma IL-10^−/−^ mice were shown to develop key symptoms of severe campylobacteriosis, such as acute enterocolitis with bloody diarrhea and wasting and pro-inflammatory immune responses that do not only affect the intestinal tract, but also extra-intestinal organs including the livers, kidneys, lung, and the systemic circulation [32]. Our recent study underscored the suitability of the hma IL-10^−/−^ mouse model to test the disease-alleviating effects of defined molecules such as carvacrol, for instance, during acute campylobacteriosis [37]. This prompted us to unravel the disease-mitigating effects of AC when applied prophylactically to hma IL-10^−/−^ in our pre-clinical placebo-controlled trial. Therefore, we assessed (i) the gastrointestinal *C. jejuni* numbers, (ii) the clinical outcome, (iii) the histopathological including apoptotic changes, (iv) the immune cell responses in the colonic mucosa and lamina propria, (v) the intestinal and (vi) the extra-intestinal pro-inflammatory mediator secretion, and finally (vii) the changes in the gut microbiota composition following *C. jejuni* infection of hma IL-10^−/−^ mice that had been pre-treated orally with AC.

## 2. Materials and Methods

### 2.1. Ethics Statement

Every day, a survey of the clinical conditions of the animals was performed. All mice studies were conducted in compliance with the 2010/63/EU European Animal Welfare Guidelines, after having received permit from the local ethics commission (“Landesamt für Gesundheit und Soziales”, LaGeSo, Berlin; registration number G0104/19).

### 2.2. Mice, Murine Commensal Gut Microbiota Eradication

**Figure 1:**
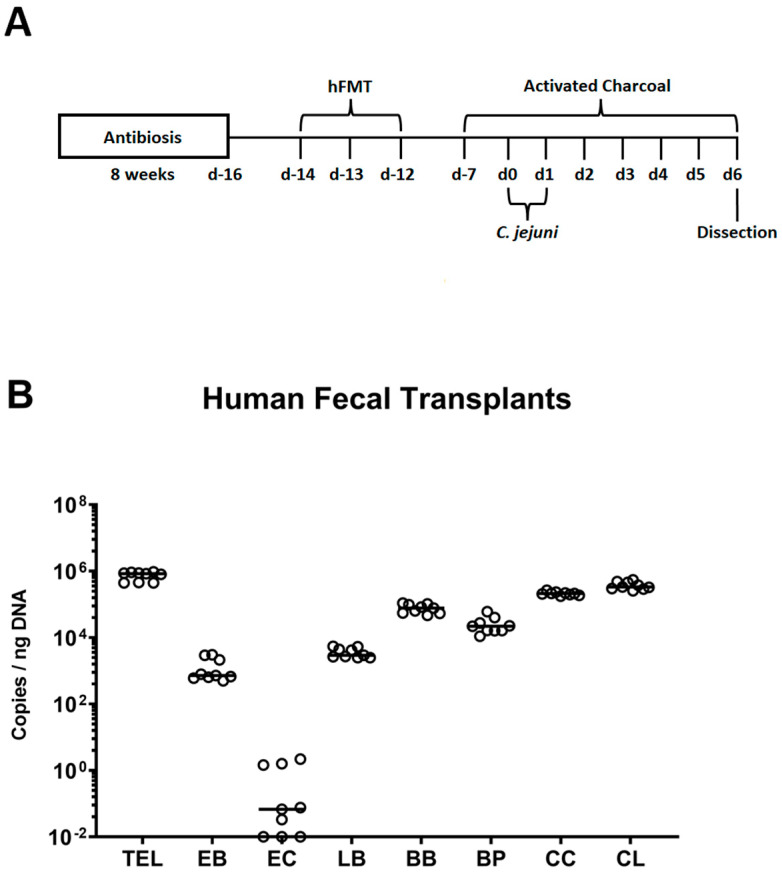
Experimental timeline and microbiota composition of human fecal donor suspensions. (**A**) Conventional IL-10^−/−^ mice were pre-treated with antibiotics for 8 weeks to deplete the commensal murine gut microbiota. Two days prior to human fecal microbiota transplantation (hFMT), the antibiotics were replaced by autoclaved tap water (i.e., on day (d)-16). The secondary abiotic IL-10^−/−^ mice were orally subjected to human FMT on d-14, d-13, and d-12. Starting on d-7, human microbiota-associated (hma) IL-10^−/−^ mice were prophylactically challenged with activated charcoal (AC) via the drinking water (*ad libitum*), infected with *Campylobacter jejuni* strain 81–176 on d-0 and d-1 by gavage, and finally sacrificed on d-6. (**B**) The microbiota composition of the human fecal transplants was quantitatively assessed by culture-independent, 16S rRNA-based methodology (see methods). Medians (black bars) are indicated and the total eubacterial loads (TEL), enterobacteria (EB), enterococci (EC), lactobacilli (LB), bifidobacteria (BB), *Bacteroides/Prevotella* species (BP), *Clostridium coccoides* (CC), and *Clostridium leptum* (CL) groups are expressed as gene copies per ng DNA. Data were pooled from three independent experiments.

Specific pathogen-free and hygienic conditions were followed during the generation and housing of IL-10^−/−^ mice (C57BL/6j background) at the Forschungsinstitute für Experimentelle Medizin, Charité—Universitätsmedizin Berlin, Germany. Mice were maintained in experimental semi-barriers in autoclaved cages with filter tops. They were given free access to sterile tap water and regular chow diet (ssniff R/M-H, V1534-300, Sniff, Soest, Germany). Three-week-old male and female mice were given ampicillin plus sulbactam (2 g/L and 1 g/L, respectively; Dr. Friedrich Eberth Arzneimittel, Ursensollen, Germany) via the drinking water (two months, *ad libitum*) in order to eradicate the commensal murine intestinal flora. Fecal specimens were cultured in enrichment broths to guarantee efficient microbial depletion, as reported earlier [38]. Every secondary abiotic mouse was kept, handled, and treated aseptically. The antibiosis was stopped 48 h before the human FMT, and autoclaved water (*ad libitum*) was used in its place (Figure 1A).

### 2.3. Transplantation of Human Fecal Microbiota

The secondary abiotic mice were orally challenged with human FMTs beginning 14 days before the *C. jejuni* infections (i.e., on days −14, −13, and −12; Figure 1A). This was conducted in order to introduce a complex commensal human intestinal microbiota into the microbiota-depleted murine host by gavage (0.3 mL volume). Prior to this, fecal donor specimens, which had been obtained from five healthy individuals (all samples free of enteropathogenic bacteria, viruses, and parasites) and stored at −80 °C, were thawed and resuspended in sterile phosphate buffered saline (PBS, Thermo Fisher Scientific, Waltham, MA, USA) as outlined earlier [32]. Figure 1B displays the commensal gut microbial loads of the donor suspensions utilized in human FMTs.

### 2.4. Preventive Measures

Sigma-Aldrich (Munich, Germany) supplied the activated charcoal, which was then dissolved in autoclaved drinking water (concentration of 10 g/L) and applied to the mice with a daily dosage of 2.5 g per kilogram of murine body weight (*ad libitum*). Only autoclaved water was given to the placebo counterparts. Mice were subjected to oral AC prophylaxis starting a week prior *C. jejuni* infection (Figure 1A).

### 2.5. Infection

After thawing from frozen stocks kept at −80 °C, *C. jejuni* strain 81–176 bacteria were cultivated for a minimum of 2 days at 37 °C in a microaerophilic environment on selective karmali agar plates (Oxoid, Wesel, Germany). As previously described [33], grown bacteria were transferred to sterile PBS (Thermo Fisher Scientific, Waltham, MA, USA) with sterile cotton swabs to create an inoculum of 10^9^ bacterial cells. On days 0 and 1, hma IL-10^−/−^ mice (12-week-old age- and sex-matched littermates) were gavaged for infection with 10^9^ colony-forming units (CFU) of the pathogen (in 0.3 mL volume).

### 2.6. Pathogen Burdens

The numbers of live *C. jejuni* bacteria were counted in fecal samples daily p.i. and intraluminal gastrointestinal specimens (colon, ileum, duodenum, stomach) were collected on day 6 p.i. For culture, specimens were homogenized in sterile PBS (Thermo Fisher Scientific, Waltham, MA, USA), diluted aliquots grown on karmali agar plates (Oxoid, Wesel, Germany) for minimum 2 days at 37 °C under microaerophilic conditions, and *C. jejuni* was quantitated by counting the CFU [33]. One hundred CFU per gram of fecal material was the detection limit for viable pathogens.

### 2.7. Gut Microbiota Composition

The human fecal transplants and the microbiota compositions of the fecal pellets collected from the hma mice were examined both immediately prior to (i.e., day 0) and six days following *C. jejuni* infection. 16S rRNA-based techniques were used to quantitatively evaluate even uncultivable commensals. Therefore, the total genomic DNA was extracted from the corresponding fecal specimen, and the predominant bacterial groups in the human gut microbiota were identified by quantitative real-time polymerase chain reaction with species-, genera-, and group-specific 16S rRNA primers (Tib MolBiol, Berlin, Germany) as outlined in detail earlier [38,39].

### 2.8. Assessment of Clinical Outcome

The clinical outcomes of the animals were recorded using clinical campylobacteriosis scores for wasting symptoms, diarrhea, and fecal blood both before and every day following the infection with *C. jejuni* (Table 1).

### 2.9. Sampling

Animals were necrotized upon carbon dioxide asphyxiation on day 6 p.i.. Luminal content from the stomach, duodenum, ileum, and colon, as well as tissue specimens from the distal large intestines, were taken aseptically.

### 2.10. Histopathology

Tissue specimens from the distal large intestines were promptly preserved in 5% formalin, embedded in paraffin, and cut into 5-µm thin slices for hematoxylin and eosin (H&E) staining. In order to record the degree of histological alterations in the colonic mucosa and lamina propria, the corresponding biopsies were graded under light microscopy (100-times magnification) (Table 2).

### 2.11. Immunohistochemistry

3-µm-thin slices of distal large intestinal paraffin specimens were stained with distinct antibodies to quantitate apoptotic epithelial cells, monocytes/macrophages, T lymphocytes, and B lymphocytes (Table 3).

### 2.12. Mediators of Inflammation

Distal large intestinal biopsies that had been cut lengthwise were cleaned from fecal matter by rinsing in PBS. In culture supernatants of corresponding tissue specimens (approximately 1 cm^2^), tumor necrosis factor-alpha (TNF-α), interleukin-6 (IL-6), and interferon-gamma (IFN-γ) concentrations were measured using the Mouse Inflammation Cytometric Bead Assay (CBA) in a BD FACSCanto II flow cytometer (both from BD Biosciences, Heidelberg, Germany) following an 18-h incubation period at 37 °C. By using the Griess reaction, colonic nitric oxide concentrations were assessed.

### 2.13. Statistics

GraphPad Prism (version 9; San Diego, CA, USA) was used to compute medians and significance levels following the pooling of data from three separate intervention trials. A survey on data set normalization was conducted using the Anderson–Darling test. Pairwise comparisons of normally distributed and non-normally distributed values were performed using the Student’s *t*-test and the Mann–Whitney test, respectively. The one-way ANOVA with Tukey post-correction (for normally distributed values) and the Kruskal–Wallis test with Dunn’s post-correction (for non-normally distributed values) were applied for multiple comparisons. Significant two-sided probability (*p*) values were defined as <0.05.

## 3. Results

### 3.1. Engraftment of Human Fecal Transplants in Murine Recipients

**Figure 2:**
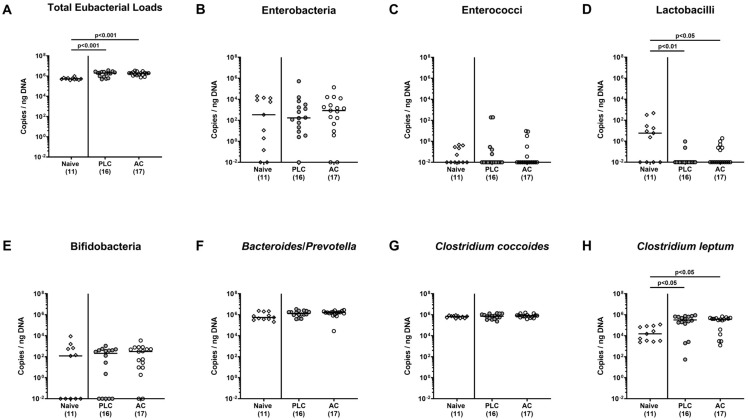
Engraftment of human fecal transplants in murine recipients. Two weeks prior to *C. jejuni* infection, (with respect to the gut microbiota) “humanized” mice were generated by oral human fecal microbiota transplantation (hFMT) of secondary abiotic IL-10^−/−^ mice on day (d)-14, d-13, and d-12 and subjected to oral activated charcoal (AC, white circles) or placebo (PLC; grey circles) prophylaxis via the drinking water starting on d-7. The engraftment of the human fecal transplants was assessed in mice immediately before *C. jejuni* infection (i.e., on d-0) by culture-independent, 16S rRNA-based methods. Untreated “humanized” mice without subsequent infection served as controls (naive). The (**A**) total eubacterial loads, (**B**) enterobacteria, (**C**) enterococci, (**D**) lactobacilli, (**E**) bifidobacteria, (**F**) *Bacteroides/Prevotella* species (spp.), (**G**) *Clostridium coccoides*, and (**H**) *Clostridium leptum* (CL) groups are expressed as gene copies per ng DNA. Medians (black bars) and numbers of bacteria-positive mice out of the total cohort (in parentheses) are indicated from three pooled independent experiments.

First, we tested the engraftment of the human fecal transplants in the microbiota-depleted murine recipients two weeks following oral human FMTs and, hence, a week after the start of the oral AC versus PLC prophylaxis (Figure 1). Therefore, we obtained fecal pellets immediately prior to *C. jejuni* infection and quantitatively assessed the fecal microbiota composition including fastidious and uncultivable commensal gut bacteria. Our molecular analyses revealed comparable fecal gene numbers of facultative and obligate anaerobic bacterial taxa in prophylactically treated and untreated hma IL-10^−/−^ mice on day 0 (not significant (n.s.); Figure 2). Hence, the fecal microbiota in human gut microbiota recipient mice was comparable immediately before *C. jejuni* infection irrespective of the applied prophylactic regimen.

### 3.2. Activated Charcoal Prophylaxis and the Gastrointestinal Pathogen Loads following C. jejuni Infection of hma IL-10^−/−^ Mice

**Figure 3:**
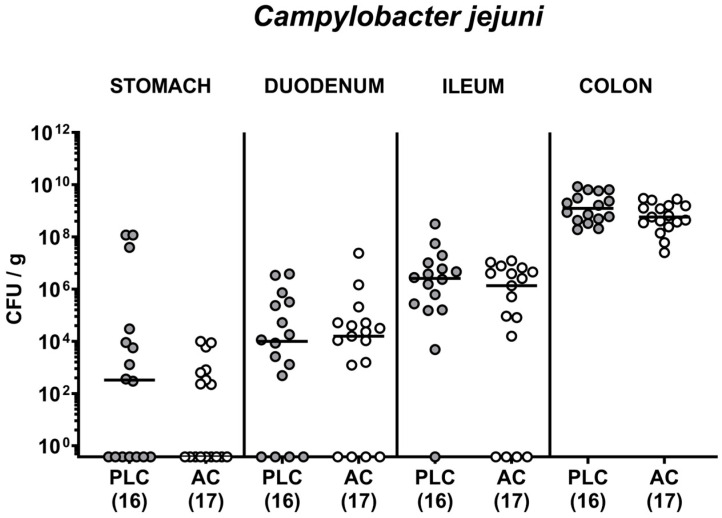
Activated charcoal prophylaxis and the gastrointestinal pathogen loads following *C. jejuni* infection of human gut microbiota-associated IL-10^−/−^ mice. Humanized IL-10^−/−^ mice were orally challenged with activated charcoal (AC; white circles) or placebo (PLC; grey circles) via the drinking water starting 7 days prior to *C. jejuni* infection on days 0 and 1. On day 6 post-infection, the *C. jejuni* bacteria were cultured from luminal samples derived from the stomach, duodenum, ileum, and colon and expressed as colony-forming units per gram (CFU/g). Individual data pooled from three experiments, medians (black bars), and the numbers of culture-positive mice out of the total number of analyzed animals (in parentheses) are shown.

Then we asked whether the AC prophylactic measures had an impact on the pathogen loads in *C. jejuni* infected hma mice. Our cultural analyses over time post-infection revealed median *C. jejuni* numbers of approximately 10^8^ viable bacteria per gram feces taken from AC and placebo challenged mice (n.s.; Supplemental Figure S1). Upon sacrifice on day 6 p.i., irrespective of the prophylactic regimen, comparable pathogen numbers were cultured in luminal samples derived from defined gastrointestinal parts with the highest counts in the colon (n.s. versus placebo; Figure 3). Hence, the oral AC prophylaxis did not affect the gastrointestinal pathogen burdens in *C. jejuni* infected hma mice.

### 3.3. Activated Charcoal Prophylaxis and Clinical Outcome of hma IL-10^−/−^ Mice following C. jejuni Infection

**Figure 4:**
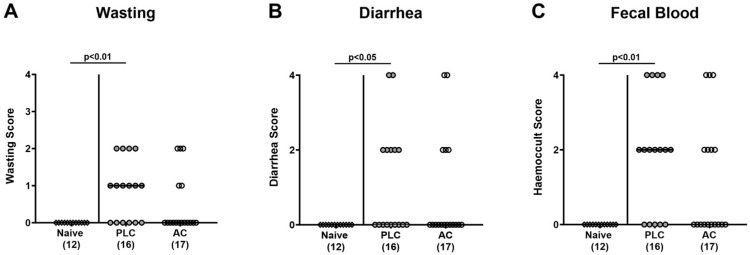
Activated charcoal prophylaxis and clinical outcome of human gut microbiota-associated IL-10^−/−^ mice following *C. jejuni* infection. Humanized IL-10^−/−^ mice were orally challenged with activated charcoal (AC; white circles) or placebo (PLC; grey circles) via the drinking water starting 7 days prior to *C. jejuni* infection on days 0 and 1. On day 6 post-infection, the clinical outcome of infected mice was quantitated with a campylobacteriosis score assessing (**A**) wasting, (**B**) diarrhea, and (**C**) fecal blood. Naive hma IL-10^−/−^ mice (white diamonds) were used as untreated and non-infected controls. The medians (black bars), the numbers of mice included from three independent experiments (in parentheses), and the significance levels (*p* values) determined by the Kruskal–Wallis test and Dunn’s post-correction are shown.

Next, we surveyed the impact of oral AC prophylaxis on the course of *C. jejuni* induced disease. Therefore, we scored the severity of the distinct campylobacteriosis symptoms over time following infection, such as wasting, diarrhea, and fecal blood. *C. jejuni* infection resulted in enhanced wasting symptoms in mice from the placebo as opposed to the AC cohort on days 3, 4, and 5 p.i. (*p* < 0.05–0.01 versus naive; Supplemental Figure S2), whereas no differences were found for severities of diarrheal symptoms at respective time points (n.s. versus naive, Supplemental Figure S3). On days 4 and 5 p.i., however, fecal blood scores were exclusively increased in placebo control mice (*p* < 0.05 versus naive; Supplemental Figure S4). When assessing the clinical outcome at the end of the observation period on day 6 p.i., scores for wasting symptoms, diarrhea, and fecal blood were increased in placebo controls only (*p* < 0.05–0.01 versus naive), whereas mice from the AC prophylaxis cohort exhibited basal values (n.s. versus naive; n.s. versus placebo; Figure 4). Hence, clinical signs of campylobacteriosis were mitigated upon oral AC prophylaxis.

### 3.4. Activated Charcoal Prophylaxis and Microscopic Inflammatory Changes in the Colon of hma IL-10^−/−^ Mice following C. jejuni Infection

**Figure 5:**
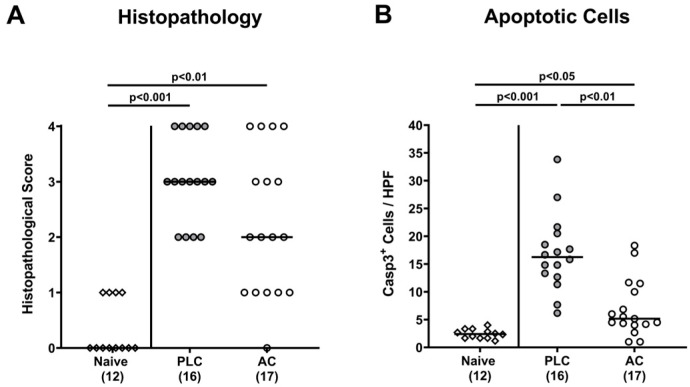
Activated charcoal prophylaxis and microscopic inflammatory changes in the colon of human gut microbiota-associated IL-10^−/−^ mice following *C. jejuni* infection. Humanized IL-10^−/−^ mice were orally challenged with activated charcoal (AC; white circles) or placebo (PLC; grey circles) via the drinking water starting 7 days prior to *C. jejuni* infection on days 0 and 1. On day 6 post-infection, both, the (**A**) histopathology was scored and the (**B**) numbers of cleaved caspase-3 positive (Casp3^+^) apoptotic colonic epithelial cells were enumerated in colonic paraffin sections (average numbers out of 6 representative high-power fields (HPF, 400-times magnification) per mouse). Naive hma IL-10^−/−^ mice (white diamonds) were used as untreated and non-infected controls. The medians (black bars), the numbers of mice included from three independent experiments (in parentheses) and the significance levels (*p* values) determined by the Kruskal–Wallis test and Dunn’s post-correction are shown.

We assessed the microscopic inflammatory sequelae *of C. jejuni* infection upon AC prophylaxis. Therefore, we scored the histopathological changes in the colonic mucosa and observed a trend towards lower histopathological scores in AC as compared to placebo treated mice on day 6 p.i. (n.s. due to high standard deviations; Figure 5A). In addition, we counted the numbers of apoptotic colonic epithelial cells after immunohistochemical staining of colonic paraffin sections with an antibody against cleaved caspase-3. On day 6 p.i., mice from the placebo control group displayed approximately 6-fold increased numbers of median apoptotic colonic epithelial cells (*p* < 0.001 versus naive; Figure 5B), whereas these increases were far less pronounced (i.e., approximately 2-fold; *p* < 0.05 versus naive) in AC treated mice (*p* < 0.01 versus placebo; Figure 5B). Hence, oral AC prophylaxis could effectively dampen *C. jejuni* induced apoptotic cell responses in the colon.

### 3.5. Activated Charcoal Prophylaxis and Immune Cell Responses in the Colon of hma IL-10^−/−^ Mice following C. jejuni Infection

**Figure 6:**
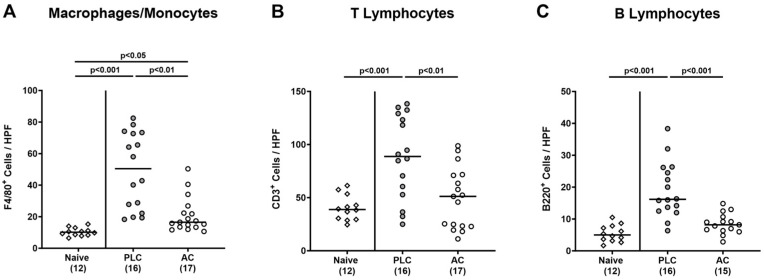
Activated charcoal prophylaxis and immune cell responses in the colon of human gut microbiota-associated IL-10^−/−^ mice following *C. jejuni* infection. Humanized IL-10^−/−^ mice were orally challenged with activated charcoal (AC; white circles) or placebo (PLC; grey circles) via the drinking water starting 7 days prior to *C. jejuni* infection on days 0 and 1. On day 6 post-infection, (**A**) macrophages and monocytes (F4/80^+^), (**B**) T lymphocytes (CD3^+^), and (**C**) B lymphocytes (B220^+^) were enumerated in colonic paraffin sections (average numbers out of 6 representative high-power fields (HPF, 400-times magnification) per mouse). Naive hma IL-10^−/−^ mice (white diamonds) were used as untreated and non-infected controls. The medians (black bars), the numbers of mice included from three independent experiments (in parentheses), and the significance levels (*p* values) determined by the Kruskal–Wallis test and Dunn’s post-correction are shown.

We tested for potential immune-modulatory effects of oral AC prophylactic measures during acute campylobacteriosis. Therefore, colonic paraffin sections were stained with antibodies against surface markers of distinct innate and adaptive immune cell subsets. Whereas *C. jejuni* infection was accompanied by increased numbers of F4/80^+^ macrophages and monocytes in the colonic mucosa and lamina propria on day 6 p.i. (*p* < 0.05–0.001 versus naive), these increases were less prominent in AC as compared to placebo treated mice (*p* < 0.01; Figure 6A). Furthermore, elevated numbers of CD3^+^ T lymphocytes and of B220^+^ B lymphocytes were detected in the colonic mucosa and lamina propria of placebo control mice only (*p* < 0.01–0.001 versus naive), whereas respective adaptive immune cell populations did not exceed basal values in AC treated mice on day 6 p.i. (n.s. versus naive; Figure 6B,C). Hence, oral AC prophylaxis dampened innate and adaptive immune cell responses in the colon upon *C. jejuni* infection.

### 3.6. Activated Charcoal Prophylaxis and Pro-Inflammatory Mediator Responses in the Intestines of hma IL-10^−/−^ Mice following C. jejuni Infection

**Figure 7:**
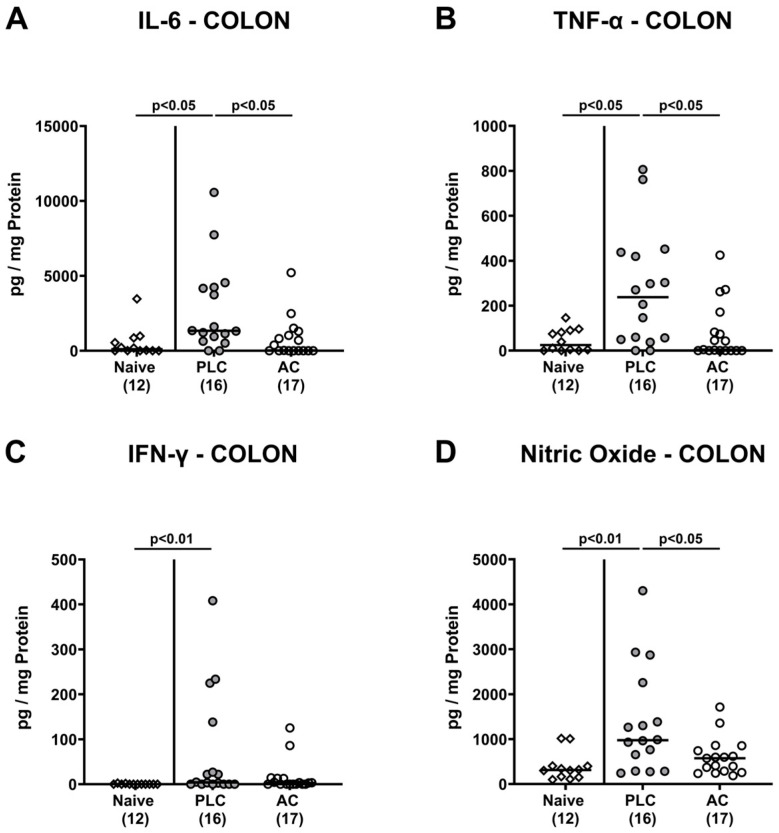
Activated charcoal prophylaxis and pro-inflammatory mediator responses in the colon of human gut microbiota-associated IL-10^−/−^ mice following *C. jejuni* infection. Humanized IL-10^−/−^ mice were orally challenged with activated charcoal (AC; white circles) or placebo (PLC; grey circles) via the drinking water starting 7 days prior to *C. jejuni* infection on days 0 and 1. (**A**) IL-6, (**B**) TNF-α, (**C**) IFN-γ, and (**D**) nitric oxide concentrations were measured in colonic explants taken on day 6 post-infection. Naive hma IL-10^−/−^ mice (white diamonds) were used as untreated and non-infected controls. The medians (black bars), the numbers of mice included from three independent experiments (in parentheses), and the significance levels (*p* values) determined by the Kruskal–Wallis test and Dunn’s post-correction are shown.

Then, we asked whether AC prophylaxis resulted in less *C. jejuni* induced pro-inflammatory mediator secretion in the large intestinal tract. In fact, increased concentrations of IL-6, TNF-α, IFN-γ, and nitric oxide were measured in colonic explants taken from placebo control mice only (*p* < 0.05–0.01 versus naive), whereas pro-inflammatory mediator concentrations did not differ in naive and in AC treated mice on day 6 p.i. (n.s. versus naive; Figure 7). Hence, oral AC prophylactic measures dampened *C. jejuni* induced colonic pro-inflammatory mediator secretion to basal levels.

### 3.7. Activated Charcoal Prophylaxis and Fecal Microbiota Compositions in hma IL-10^−/−^ Mice during C. jejuni Infection

**Figure 8:**
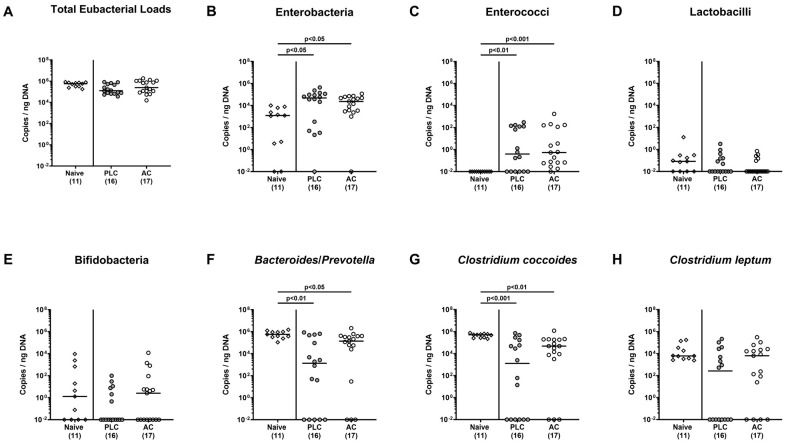
Activated charcoal prophylaxis and fecal microbiota compositions in human gut microbiota-associated IL-10^−/−^ mice during *C. jejuni* infection. Humanized IL-10^−/−^ mice were orally challenged with activated charcoal (AC; white circles) or placebo (PLC; grey circles) via the drinking water starting 7 days prior to *C. jejuni* infection on day (d)-0 and d-1. The fecal microbiota compositions were surveyed on d-6 after *C. jejuni* infection by culture-independent, molecular methods. Untreated “humanized” mice without subsequent infection served as controls (naive). The (**A**) total eubacterial loads, (**B**) enterobacteria, (**C**) enterococci, (**D**) lactobacilli, (**E**) bifidobacteria, (**F**) *Bacteroides/Prevotella* species, (**G**) *Clostridium coccoides*, and (**H**) *Clostridium leptum* groups are expressed as copies per ng DNA. The medians (black bars), the numbers of mice with bacteria-positive detection out of the total number of analyzed animals (in parentheses), and the significance levels (*p* values) determined by the Kruskal–Wallis test and Dunn’s post-correction are indicated from three pooled independent experiments.

Finally, we addressed whether AC prophylaxis had an impact on potential human gut microbiota shifts during *C. jejuni* infection of transplanted mice. Our comprehensive molecular analyses (Figure 8) revealed lower total eubacterial loads in fecal pellets collected from placebo and AC treated mice on day 6 p.i. when compared to day 0 (*p* < 0.001; Figure 8A), which were paralleled by lower gene numbers of obligate anaerobic bacterial taxa such as bifidobacteria (*p* < 0.05–0.01; Figure 8E), *Bacteroides*/*Prevotella* species (*p* < 0.001; Figure 8F), *Clostridium coccoides* (*p* < 0.001; Figure 8G), and *Clostridium leptum* (*p* < 0.001; Figure 8H). Conversely, gene numbers of fecal enterobacteria increased from day 0 until day 6 following infection of both placebo and AC prophylactically treated mice (*p* < 0.05–0.01; Figure 8B). Of note, in transplanted non-infected control mice, respective fecal microbiota shifts were not observed between day 0 and day 6 (n.s.; Figure 8A,B,E–H). Hence, *C. jejuni* infection resulted in distinct fecal microbiota shift towards higher enterobacterial numbers and lower loads of obligate anaerobic species in hma mice that were independent of oral AC application, however.

## 4. Discussion

AC has long been used as remedy for diarrheal morbidities of multiple etiologies, including intoxications and infectious diseases affecting the intestinal tract, given its potent adsorptive properties [22]. In fact, AC was shown to directly bind and inactivate exotoxins from *E. coli* and *Vibrio cholerae*, for instance [40]. Data from clinical studies addressing the therapeutic and prophylactic effects of AC in *C. jejuni* enteritis are scarce, however. In our present pre-clinical intervention trial, we tested the effects of oral AC versus placebo prophylaxis in the course of campylobacteriosis in hma IL-10^−/−^ mice and found (i) comparable gastrointestinal pathogen burdens upon *C. jejuni* infection, but (ii) pronounced *C. jejuni* induced clinical signs such as wasting symptoms and bloody diarrhea in placebo as opposed to AC challenged mice, that were accompanied by (iii) less distinct apoptotic and (iv) pro-inflammatory immune responses in the colon of the latter versus the former. At the end of the experiments, (v) the fecal microbiota compositions in *C. jejuni* infected hma IL-10^−/−^ mice did not differ between the two oral prophylaxis cohorts.

The results of comparable *C. jejuni* loads in the gastrointestinal tracts of mice from the AC and placebo groups are not unexpected given that the direct antimicrobial potency of AC is regarded as rather minor, if it exists at all [41]. An anti-*C. jejuni* effect of AC is unlikely since *Campylobacter*-selective growth media (including the here used karmali agar) are supplemented with charcoal to optimize enteropathogenic growth [42]. Despite comparable *C. jejuni* numbers in the gastrointestinal lumen of infected mice, the better clinical outcome upon AC prophylaxis might have been at least in part due to potential binding and immobilization of *C. jejuni* by the compound counteracting further bacterial invasion and subsequent activation of the host immune system. This hypothesis is supported by a previous study showing that AC could bind toxin-producing *E. coli* in vitro [23]. The disease-mitigating effects of AC prophylaxis could also be observed on the microscopic level given less distinct colonic epithelial cell apoptosis and attenuated recruitment of both innate and adaptive immune cell populations to the infected colonic mucosa and lamina propria of hma IL-10^−/−^ mice. Remarkably, the concentrations of pro-inflammatory mediators including IL-6, TNF-α, IFN-γ, and nitric oxide measured in the colon of mice from the AC prophylaxis cohort did not differ from naive (i.e., uninfected and untreated) “humanized” control mice, which, in turn, resulted in diminished *C. jejuni* induced oxidative stress to the intestinal epithelia. Our presented results are supported by our previous study [43] where we tested the therapeutic effects of AC in another acute murine campylobacteriosis model and initiated oral AC treatment (with the same concentration) two days after *C. jejuni* infection of secondary abiotic IL-10^−/−^ mice lacking commensal gut bacteria. Notably, the shorter therapeutic oral AC regimen resulted in an improved clinical outcome, in less severe colonic epithelial apoptosis, and in diminished pro-inflammatory mediator secretion in the intestinal tract of *C. jejuni* infected mice on day 6 p.i. [43]. Since the *C. jejuni*-derived endotoxin LOS is a key player in mediating the TLR-4-driven hyperactivation of the immune cascade mounting in acute campylobacteriosis [44], it is tempting to speculate that the disease-mitigating effects of exogenous AC might have been due to the direct binding of the enteropathogenic endotoxin as also shown for LPS before as well as of binding and inactivation of the secreted pro-inflammatory mediators in vitro [31,45,46]. To date, experimental in vivo data regarding the anti-inflammatory effects of exogenous AC are very limited. Afrin et al. reported that following oral treatment with the charcoal supplement Le Carbone, mice suffered less distinctly from acute dextran sulfate sodium induced colitis as indicated by improved clinical conditions, down-regulated intestinal expression of pro-inflammatory mediators including TNF-α, and less distinct apoptotic cell responses [47].

Furthermore, we surveyed the fecal gut microbiota compositions during acute campylobacteriosis in mice from the prophylaxis groups. Our quantitative culture-independent analyses revealed that the fecal microbiota in human microbiota recipient mice was comparable immediately before *C. jejuni* infection irrespective of the applied prophylactic regimen, indicative for a comparable engraftment of the human fecal transplant in recipient secondary abiotic mice. At the end of the experiment, *C. jejuni* infected mice exhibited higher enterobacterial numbers, but lower loads of obligate anaerobic species including bifidobacteria, *Bacteroides*/*Prevotella* species, *C. coccoides* and *C. leptum* groups when compared to naive control animals that were, however, independent of the prophylactic oral AC application. The here observed *C. jejuni* induced fecal microbiota shifts could be confirmed by our previous studies showing increased colonic enterobacterial counts but decreased obligate anaerobic bacterial numbers during acute campylobacteriosis in hma IL-10^−/−^ mice [32,37]. Given that direct antibiotic effects of AC have not been described to date, the risk of dysbiosis and of the development of antibacterial resistance upon AC treatment can be considered as negligible.

## 5. Conclusions

The used hma IL-10^−/−^ mice constitute a valuable acute campylobacteriosis model to dissect the interplay between distinct prophylactically or therapeutically applied compounds, the enteropathogen, host immunity, and human gut commensals *in vivo*. Our pre-clinical placebo-controlled intervention study provides evidence that prophylactic oral application of AC mitigates acute campylobacteriosis in hma IL-10^−/−^ mice and could open novel avenues for both, the prevention and treatment of human *C. jejuni* infection due to its multi-facetted modes of action including binding and inactivation of viable pathogens, of endotoxins, and of pro-inflammatory mediators.

Since the severity of *C. jejuni* induced enteritis is positively correlated to the risk of the development of post-infectious autoimmune morbidities [19], it is tempting to speculate that prophylactic oral AC application to humans with increased exposure to *Campylobacter* species might not only mitigate acute enteritis, but also reduce the risk for post-infectious collateral damages such as GBS.

Overall, AC constitutes an inexpensive over-the-counter treatment option with a non-toxic, and relatively safe compound given if at all, rather negligible adverse effects including interfering with absorption of vitamins and other medications, for instance [26]. Finally, AC might be considered for the prophylaxis and/or treatment of other food-borne enteropathogenic diseases.

## Figures and Tables

**Table 1:** Clinical scores (maximum 12 points).

Clinical Aspect	Scores
Wasting symptoms	0: normal 1: ruffled fur 2: less locomotion 3: isolation 4: severely compromised locomotion, pre-final aspect
Stool consistency	0: formed feces 2: pasty feces 4: liquid feces
Fecal blood	0: no blood 2: microscopic detection of blood by the Guajac method using Haemoccult, Beckman Coulter/PCD, Germany 4: macroscopic blood visible

**Table 2:** Histopathological scores.

Score 0	Normal epithelium without inflammatory cell infiltrates
Score 1	Minimal inflammatory cell infiltrates in the mucosa with intact epithelium
Score 2	Mild inflammatory cell infiltrates in the mucosa and submucosa with mild hyperplasia and mild goblet cell loss
Score 3	Moderate inflammatory cell infiltrates in the mucosa and submucosa with moderate goblet cell loss
Score 4	Marked inflammatory cell infiltration into the mucosa and submucosa with marked goblet cell loss, multiple crypt abscesses, and crypt loss

**Table 3:** Primary antibodies for in situ immunohistochemical analyses.

Cells	Primary Antibody
Apoptotic epithelial cells	cleaved caspase-3 (Asp175, Cell Signaling, Beverly, MA, USA, 1:200)
Macrophages/monocytes	F4/80 (no. 14-4801, clone BM8, eBioscience, San Diego, CA, USA, 1:50)
T lymphocytes	CD3 (no. N1580, Dako, Glostrup, Denmark, 1:10)
B lymphocytes	B220 (no. 337 14-0452-81, eBioscience; 1:200)

## Data Availability

The corresponding author provides the data from this study on request.

## Supplementary Materials

The following supporting information can be downloaded at: https://www.mdpi.com/article/10.3390/biom14020141/s1, Figure S1. Activated charcoal prophylaxis and the intestinal pathogen loads over time following *C. jejuni* infection of human gut microbiota-associated IL-10^−/−^ mice. Figure S2. Activated charcoal prophylaxis and wasting symptoms in human gut microbiota-associated IL-10^−/−^ mice over time following *C. jejuni* infection. Figure S3. Activated charcoal prophylaxis and diarrheal symptoms in human gut microbiota-associated IL-10^−/−^ mice over time following *C. jejuni* infection. Figure S4. Activated charcoal prophylaxis and fecal blood in human gut microbiota-associated IL-10^−/−^ mice over time following *C. jejuni* infection.

## References

[1] WHO Campylobacter. https://www.who.int/news-room/fact-sheets/detail/campylobacter.

[2] WHO Joint FAO/WHO Expert Meeting on the Pre- and Post-Harvest Control of Campylobacter Spp. in Poultry Meat. https://www.who.int/news-room/events/detail/2023/02/06/default-calendar/joint-fao-who-expert-meeting-on-the-pre-and-post-harvest-control-of-campylobacter-spp.-in-poultry-meat.

[3] European Food Safety Authority, European Centre for Disease Prevention and Control (2022). The European Union Summary Report on Antimicrobial Resistance in zoonotic and indicator bacteria from humans, animals and food in 2019–2020. EFSA J..

[4] Wilson D.J., Gabriel E., Leatherbarrow A.J., Cheesbrough J., Gee S., Bolton E., Fox A., Fearnhead P., Hart C.A., Diggle P.J. (2008). Tracing the source of campylobacteriosis. PLoS Genet..

[5] Fitzgerald C. (2015). Campylobacter. Clin. Lab. Med..

[6] Silva J., Leite D., Fernandes M., Mena C., Gibbs P.A., Teixeira P. (2011). *Campylobacter* spp. as a foodborne pathogen: A review. Front. Microbiol..

[7] Black R.E., Levine M.M., Clements M.L., Hughes T.P., Blaser M.J. (1988). Experimental *Campylobacter jejuni* infection in humans. J. Infect. Dis..

[8] Allos B.M. (2001). *Campylobacter jejuni* Infections: Update on emerging issues and trends. Clin. Infect. Dis..

[9] Cróinín T., Backert S. (2012). Host epithelial cell invasion by *Campylobacter jejuni: Trigger* or zipper mechanism?. Front. Cell Infect. Microbiol..

[10] Tegtmeyer N., Sharafutdinov I., Harrer A., Esmaeili D.S., Linz B., Backert S. (2021). Campylobacter Virulence Factors and Molecular Host–Pathogen Interactions. Fighting Campylobacter Infections: Towards a One Health Approach.

[11] Callahan S.M., Dolislager C.G., Johnson J.G. (2021). The host cellular immune response to infection by *Campylobacter* spp. and its role in disease. Infect. Immun..

[12] Young K.T., Davis L.M., Dirita V.J. (2007). *Campylobacter jejuni: Molecular* biology and pathogenesis. Nat. Rev. Microbiol..

[13] Butkevych E., Lobo de Sá F.D., Nattramilarasu P.K., Bücker R. (2020). Contribution of Epithelial Apoptosis and Subepithelial Immune Responses in *Campylobacter jejuni*-Induced Barrier Disruption. Front. Microbiol..

[14] Lobo de Sá F., Schulzke J.-D., Bücker R. (2021). Diarrheal mechanisms and the role of intestinal barrier dysfunction in *Campylobacter* infections. Curr. Top. Microbiol. Immunol..

[15] Finsterer J. (2022). Triggers of Guillain-Barré Syndrome: *Campylobacter jejuni* Predominates. Int. J. Mol. Sci..

[16] Nachamkin I., Allos B.M., Ho T. (1998). *Campylobacter* species and Guillain-Barre syndrome. Clin. Microbiol. Rev..

[17] Pope J.E., Krizova A., Garg A.X., Thiessen-Philbrook H., Ouimet J.M. (2007). *Campylobacter* reactive arthritis: A systematic review. Semin. Arthritis Rheum..

[18] Omarova S., Awad K., Moos V., Püning C., Gölz G., Schulzke J.-D., Bücker R. (2023). Intestinal Barrier in Post-*Campylobacter jejuni* Irritable Bowel Syndrome. Biomolecules.

[19] Mortensen N.P., Kuijf M.L., Ang C.W., Schiellerup P., Krogfelt K.A., Jacobs B.C., van Belkum A., Endtz H.P., Bergman M.P. (2009). Sialylation of *Campylobacter jejuni* lipo-oligosaccharides is associated with severe gastro-enteritis and reactive arthritis. Microbes Infect..

[20] Backert S., Tegtmeyer N., Cróinín T.Ó., Boehm M., Heimesaat M.M., Klein G. (2017). Human campylobacteriosis. Campylobacter.

[21] Dai L., Sahin O., Grover M., Zhang Q. (2020). New and alternative strategies for the prevention, control, and treatment of antibiotic-resistant *Campylobacter*. Transl. Res..

[22] Juurlink D.N. (2016). Activated charcoal for acute overdose: A reappraisal. Br. J. Clin. Pharmacol..

[23] Naka K., Watarai S., Inoue K., Kodama Y., Oguma K., Yasuda T., Kodama H. (2001). Adsorption effect of activated charcoal on enterohemorrhagic *Escherichia coli*. J. Vet. Med. Sci..

[24] Ilomuanya M., Ifudu N., Uboh C. (2011). The use of metronidazole and activated charcoal in the treatment of diarrhea caused by *Escherichia coli* 0157: H7 in an in vitro pharmacodynamic model. Afr. J. Pharm. Pharmacol..

[25] Watarai S., Koiwa M. (2008). Feeding activated charcoal from bark containing wood vinegar liquid (nekka-rich) is effective as treatment for cryptosporidiosis in calves. J. Dairy. Sci..

[26] Senderovich H., Vierhout M.J. (2018). Is there a role for charcoal in palliative diarrhea management?. Curr. Med. Res. Opin..

[27] Sergio G.C., Félix G.M., Luis J.V. (2008). Activated charcoal to prevent irinotecan-induced diarrhea in children. Pediatr. Blood Cancer.

[28] Michael M., Brittain M., Nagai J., Feld R., Hedley D., Oza A., Siu L., Moore M.J. (2004). Phase II study of activated charcoal to prevent irinotecan-induced diarrhea. J. Clin. Oncol..

[29] Hübner W.D., Moser E.H. (2002). Charcoal tablets in the treatment of patients with irritable bowel syndrome. Adv. Ther..

[30] Kurti F., Duni A., Plefka E., Çina T. (2014). Treatment of irritable bowel syndrome symptoms and the role of compounded charcoal-A multicentre study. Albanian Med. J..

[31] Nagaki M., Hughes R., Lau J., Williams R. (1991). Removal of endotoxin and cytokines by adsorbents and the effect of plasma protein binding. Int. J. Artif. Organs.

[32] Shayya N.W., Foote M.S., Langfeld L.Q., Du K., Bandick R., Mousavi S., Bereswill S., Heimesaat M.M. (2023). Human microbiota associated IL-10^−/−^ mice: A valuable enterocolitis model to dissect the interactions of *Campylobacter jejuni* with host immunity and gut microbiota. Eur. J. Microbiol. Immunol..

[33] Bereswill S., Fischer A., Plickert R., Haag L.M., Otto B., Kuhl A.A., Dasti J.I., Zautner A.E., Munoz M., Loddenkemper C. (2011). Novel murine infection models provide deep insights into the “menage a trois” of *Campylobacter jejuni*, microbiota and host innate immunity. PLoS ONE.

[34] Herzog M.K.-M., Cazzaniga M., Peters A., Shayya N., Beldi L., Hapfelmeier S., Heimesaat M.M., Bereswill S., Frankel G., Gahan C.G. (2023). Mouse models for bacterial enteropathogen infections: Insights into the role of colonization resistance. Gut Microbes.

[35] Warren H.S., Fitting C., Hoff E., Adib-Conquy M., Beasley-Topliffe L., Tesini B., Liang X., Valentine C., Hellman J., Hayden D. (2010). Resilience to bacterial infection: Difference between species could be due to proteins in serum. J. Infect. Dis..

[36] Lippert E., Karrasch T., Sun X., Allard B., Herfarth H.H., Threadgill D., Jobin C. (2009). Gnotobiotic IL-10; NF-kappaB mice develop rapid and severe colitis following *Campylobacter jejuni* infection. PLoS ONE.

[37] Foote M.S., Du K., Mousavi S., Bereswill S., Heimesaat M.M. (2023). Therapeutic Oral Application of Carvacrol Alleviates Acute Campylobacteriosis in Mice Harboring a Human Gut Microbiota. Biomolecules.

[38] Heimesaat M.M., Bereswill S., Fischer A., Fuchs D., Struck D., Niebergall J., Jahn H.-K., Dunay I.R., Moter A., Gescher D.M. (2006). Gram-negative bacteria aggravate murine small intestinal Th1-type immunopathology following oral infection with *Toxoplasma gondii*. J. Immunol..

[39] Weschka D., Mousavi S., Biesemeier N., Bereswill S., Heimesaat M.M. (2021). Survey of Pathogen-Lowering and Immuno-Modulatory Effects Upon Treatment of *Campylobacter coli*-Infected Secondary Abiotic IL-10^−/−^ Mice with the Probiotic Formulation Aviguard^®^. Microorganisms.

[40] Ramu J., Clark K., Woode G., Sarr A., Phillips T. (1997). Adsorption of cholera and heat-labile *Escherichia coli* enterotoxins by various adsorbents: An in vitro study. J. Food Prot..

[41] Rollinger Y., Dott W. (1987). Survival of selected bacterial species in sterilized activated carbon filters and biological activated carbon filters. Appl. Environ. Microbiol..

[42] Karmali M.A., Simor A., Roscoe M., Fleming P., Smith S., Lane J. (1986). Evaluation of a blood-free, charcoal-based, selective medium for the isolation of *Campylobacter* organisms from feces. J. Clin. Microbiol..

[43] Bereswill S., Mousavi S., Weschka D., Heimesaat M.M. (2021). Disease-Alleviating Effects of Peroral Activated Charcoal Treatment in Acute Murine Campylobacteriosis. Microorganisms.

[44] Mousavi S., Bereswill S., Heimesaat M.M. (2021). Murine Models for the Investigation of Colonization Resistance and Innate Immune Responses in *Campylobacter Jejuni* Infections. Curr. Top. Microbiol. Immunol..

[45] Hoffman P., Pine L., Bell S. (1983). Production of superoxide and hydrogen peroxide in medium used to culture *Legionella pneumophila*: Catalytic decomposition by charcoal. Appl. Environ. Microbiol..

[46] Bysani G.K., Shenep J.L., Hildner W.K., Stidham G.L., Roberson P.K. (1990). Detoxification of plasma containing lipopolysaccharide by adsorption. Crit. Care Med..

[47] Afrin M.R., Arumugam S., Rahman M.A., Karuppagounder V., Sreedhar R., Harima M., Suzuki H., Nakamura T., Miyashita S., Suzuki K. (2017). Le Carbone, a charcoal supplement, modulates DSS-induced acute colitis in mice through activation of AMPKα and downregulation of STAT3 and caspase 3 dependent apoptotic pathways. Int. Immunopharmacol..

