# Supplementary material for: Prophylactic Oral Application of Activated Charcoal Mitigates Acute Campylobacteriosis in Human Gut Microbiota-Associated IL-10−/− Mice
Source: Biomolecules. 2024 Jan 23;14(2):141. doi: 10.3390/biom14020141 (PMC10886519; doi:10.3390/biom14020141)
Supplement: Supplementary file 1 [file biomolecules-14-00141-s001.zip › biomolecules-2752350-supplementary.pdf]

# Supplemental Material

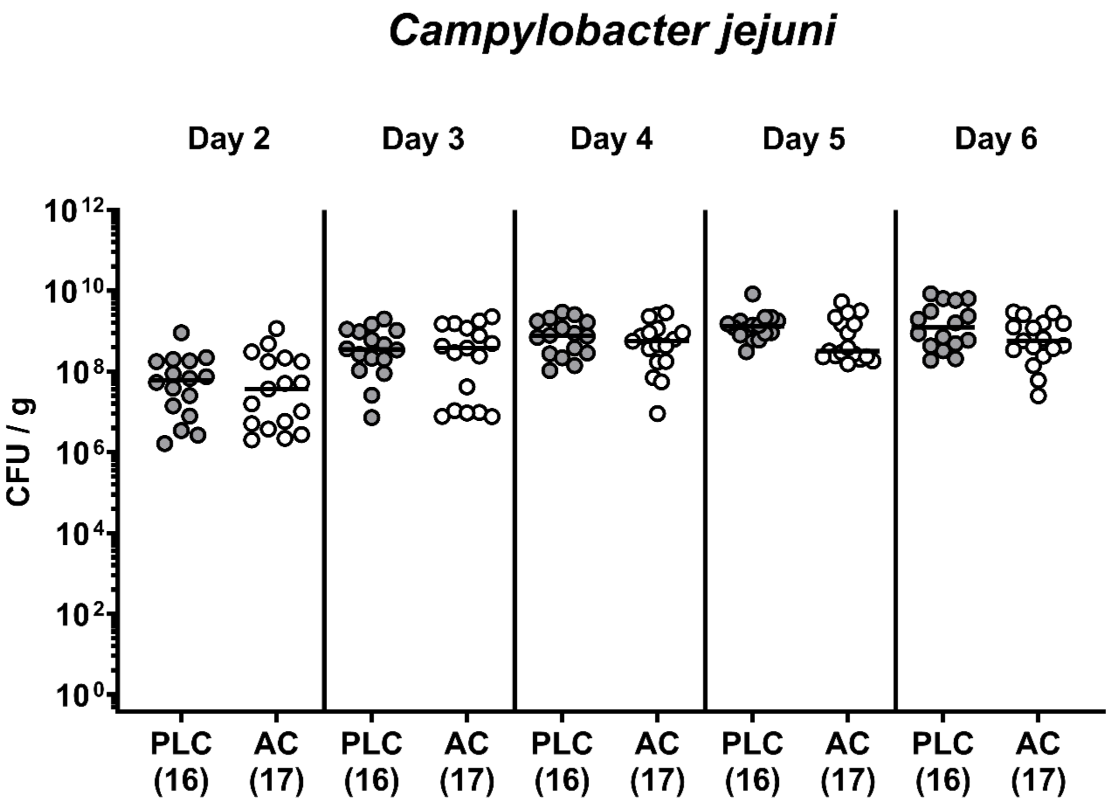


**Figure S1.** Activated charcoal prophylaxis and the intestinal pathogen loads over time following *C. jejuni* infection of human gut microbiota-associated IL-$10^{-/-}$ mice. Humanized IL-$10^{-/-}$ mice were orally challenged with activated charcoal (AC; white circles) or placebo (PLC; grey circles) via the drinking water starting 7 days prior to *C. jejuni* infection on days 0 and 1. The *C. jejuni* colonization in the intestinal tract was quantitatively assessed in fecal samples taken at defined time points post-infection (as indicated) and expressed as colony-forming units per gram (CFU/g). The medians (black bars) and the numbers of analyzed animals from three independent experiments (in parentheses) are shown.

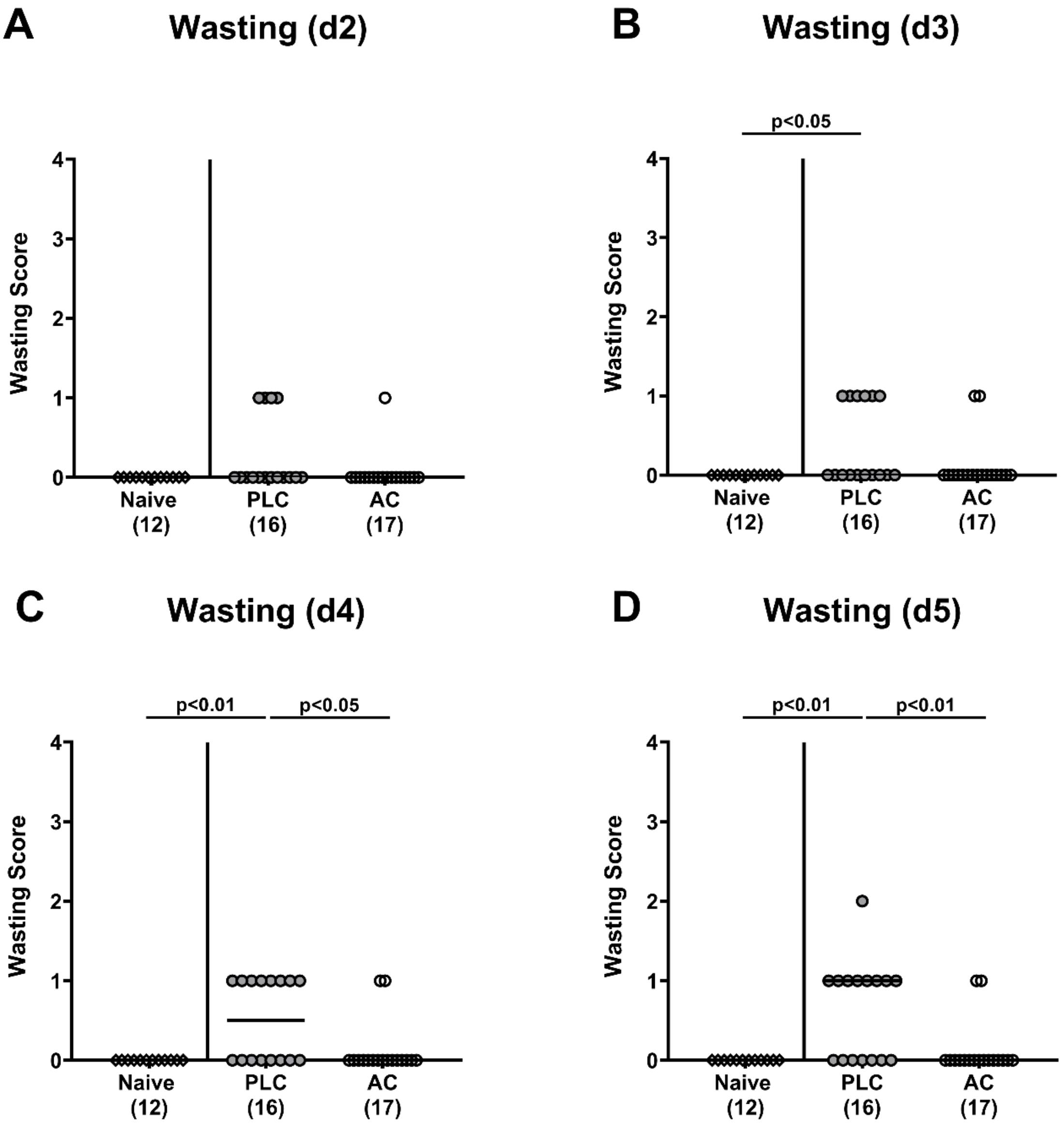


**Figure S2.** Activated charcoal prophylaxis and wasting symptoms in human gut microbiota-associated IL-10$^{-/-}$ mice over time following *C. jejuni* infection. Humanized IL-10$^{-/-}$ mice were orally challenged with activated charcoal (AC; white circles) or placebo (PLC; grey circles) via the drinking water starting 7 days prior to *C. jejuni* infection on day (d) 0 and d1. Wasting symptoms were quantitated with a defined score on (**A**) d2, (**B**) d3, (**C**) d4, and (**D**) d5 post-infection. Naive hma IL-10$^{-/-}$ mice (white diamonds) were used as untreated and non-infected controls. The medians (black bars), the numbers of mice included from three independent experiments (in parentheses), and the significance levels (p values) determined by the Kruskal–Wallis test and Dunn's post-correction are shown.

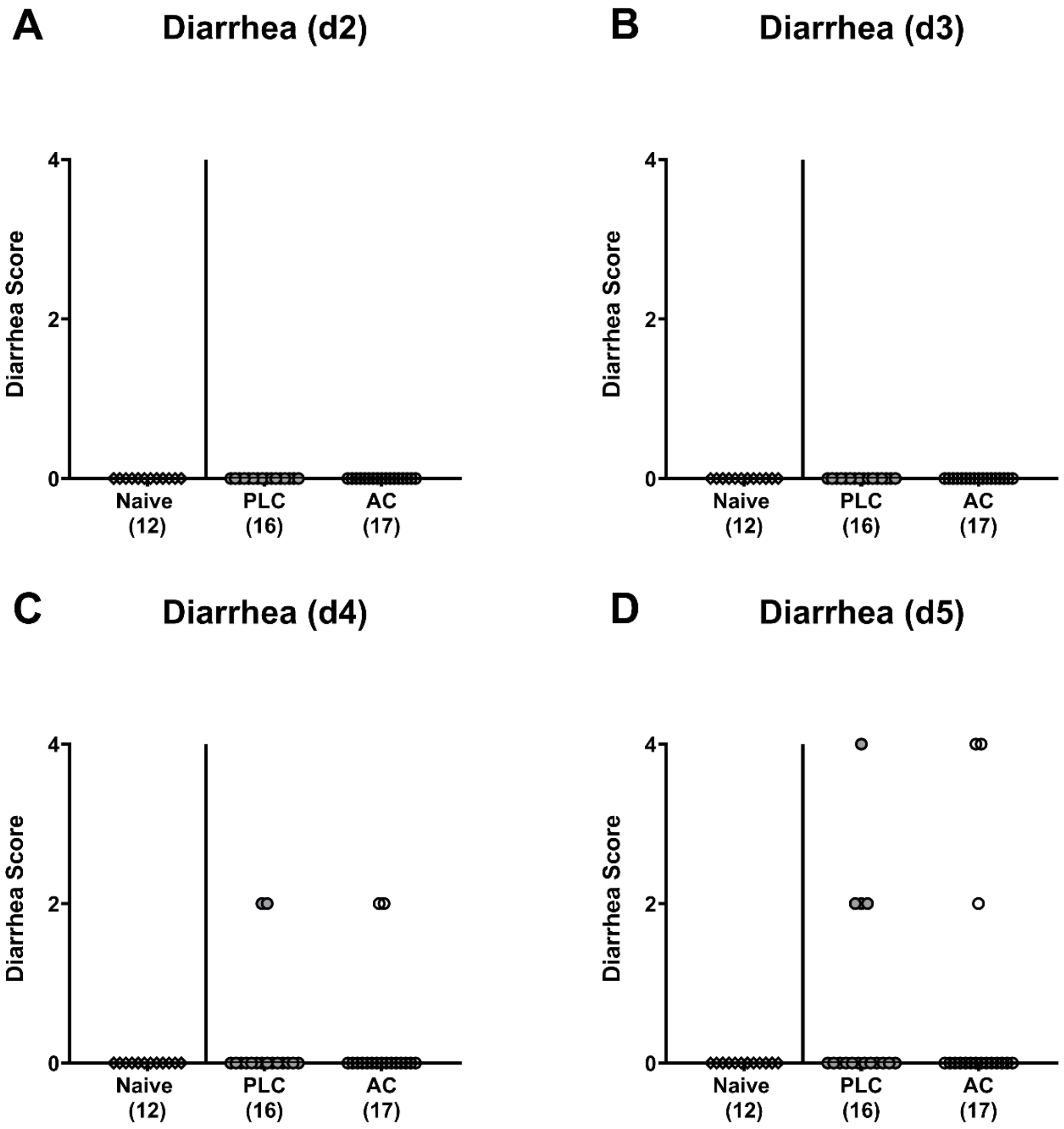


**Figure S3.** Activated charcoal prophylaxis and diarrheal symptoms in human gut microbiota-associated IL-10$^{-/-}$ mice over time following *C. jejuni* infection. Humanized IL-10$^{-/-}$ mice were orally challenged with activated charcoal (AC; white circles) or placebo (PLC; grey circles) via the drinking water starting 7 days prior to *C. jejuni* infection on day (d) 0 and d1. Diarrheal symptoms were quantitated with a defined score on (**A**) d2, (**B**) d3, (**C**) d4, and (**D**) d5 post-infection. Naive hma IL-10$^{-/-}$ mice (white diamonds) were used as untreated and non-infected controls. The medians (black bars) and numbers of mice included from three independent experiments (in parentheses) are shown.

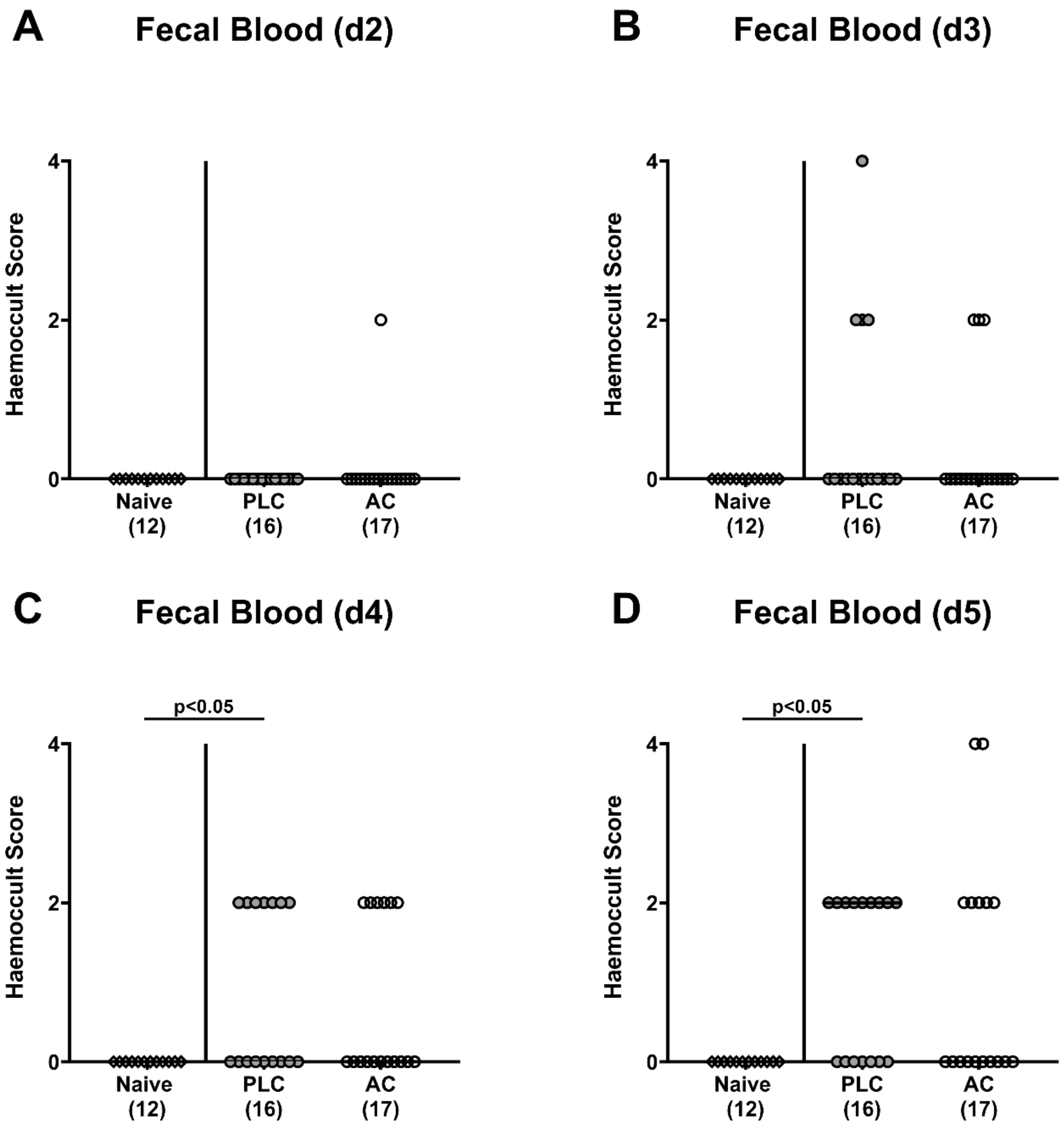


**Figure S4.** Activated charcoal prophylaxis and fecal blood in human gut microbiota-associated IL-10$^{-/-}$ mice over time following *C. jejuni* infection. Humanized IL-10$^{-/-}$ mice were orally challenged with activated charcoal (AC; white circles) or placebo (PLC; grey circles) via the drinking water starting 7 days prior to *C. jejuni* infection on day (d) 0 and d1. The abundance of fecal blood was quantitated with a defined score on (**A**) d2, (**B**) d3, (**C**) d4, and (**D**) d5 post-infection. Naive hma IL-10$^{-/-}$ mice (white diamonds) were used as untreated and non-infected controls. The medians (black bars), the numbers of mice included from three independent experiments (in parentheses), and the significance levels (p values) determined by the Kruskal–Wallis test and Dunn's post-correction are shown.
